# Residents living in communities with higher civic participation report higher self-rated health

**DOI:** 10.1371/journal.pone.0241221

**Published:** 2020-10-23

**Authors:** Moeko Noguchi-Shinohara, Kohei Hirako, Hiromasa Tsujiguchi, Tomoya Itatani, Kiyoko Yanagihara, Hikaru Samuta, Hiroyuki Nakamura

**Affiliations:** 1 Department of Neurology and Neurobiology of Aging, Kanazawa University Graduate School of Medical Sciences, Kanazawa University, Kanazawa, Japan; 2 Department of Preemptive Medicine for Dementia, Kanazawa University Graduate School of Medical Sciences, Kanazawa University, Kanazawa, Japan; 3 Industry-Academia-Government Collaboration / Intellectual Property Promotion Group, Organization of Frontier Science and Social Co-creation Initiative, Kanazawa University, Kanazawa, Japan; 4 Department of Public Health, Kanazawa University Graduate School of Advanced Preventive Medical Sciences, Kanazawa, Japan; 5 Kanazawa University Advanced Preventive Medical Sciences Research Center, Kanazawa, Japan; 6 Faculty of Health Sciences, Institute of Medical, Pharmaceutical and Health Sciences, Kanazawa University, Kanazawa, Japan; 7 Faculty of Economics and Management, Institute of Human and Social Sciences, Kanazawa University, Kanazawa, Japan; 8 Department of Environmental and Preventive Medicine, Kanazawa University Graduate School of Medical Sciences, Kanazawa University, Kanazawa, Japan; Ehime University Graduate School of Medicine, JAPAN

## Abstract

It has been shown that community-level social capital may affect residents’ health. The present mixed ecological study assesses the evidence for an association between the community-level social capital and the individual level of self-rated health. The Hakui City Health Interview Survey targeted 15,242 people aged 40 years and older from 11 communities. Among them, 6578 residents responded to the questionnaire (response rate, 43.2%). We examined whether the community-level social capital (general trust, norm, and civic participation) was associated with the individual level of self-rated health. Overall, 1919 (29.1%) answers of self-rated poor health were identified. Community-level civic participation was negatively associated with poor self-rated health after adjusting for individual demographic factors, individual social capitals, and community-level economic status, whereas community-level general trust, and norm were not significant. The findings suggest the importance of fostering communities with high civic participation to reduce the poor health status of residents.

## Introduction

Social capital has been considered an important determinant of population health [1]. Recently, a considerable number of studies has shown associations between social capital and health at an individual level [2]. However, on a community-level, the relationships between social capital and health were inconsistent, especially in the rural areas [2–12]. Some studies have demonstrated significant relationships between community- and individual-level social capital and self-rated health only in the urban areas, but not in the rural areas [10, 11]. Moreover, one study has reported that individual-level social capital influenced the self-rated health in both urban and rural areas [12].

In Japan, the population is dense in large cities, whereas depopulation is progressing in rural settings. In Hakui city, the population is declining and aging. The peak population of Hakui city was approximately 29,000 in 1985, and the population in 2019 was 21,974, the current population is three-quarters of its peak. Additionally, the proportion of the population aged 65 years or older was 37.6% in 2019. The Hakui City Health Interview Survey was conducted to investigate the attitude of Hakui citizens toward their self-rated health and the cognitive and structural components of social capital. It has been shown that global self-rated health is an independent predictor of mortality, regardless of other medical, behavioral or psychosocial factors [13]. Social capital is measured in terms of cognitive components, such as general trust and norms, and structural components, such as civic participation. It remains unclear whether community-level social capital has a beneficial effect on health, especially in a town where the population is declining and aging is progressing, whether it had positive effects, and whether all or any aspect of social capital is effective.

The aim of this mixed ecological study is to examine the association between community-level social capital and individual level of self-rated health in a rural town where the population has declined and aging has progressed.

## Materials and methods

The Hakui City Health Interview Survey was conducted by Kanazawa University and the municipal government of Hakui city, Ishikawa prefecture, Japan, in September 2019. Self-administered questionnaires were mailed to all residents of Hakui city aged 40 years or over. The total population of Hakui city in 2019 was 21,975 (10,405 men and 11,570 women) and the number of people aged ≥40 years was 15,242 (7008 men and 8234 women); among them, 6578 residents responded to the questionnaire (2912 men, 3629 women, and 37 unknown; participation rate, 43.2%).

### Ethical issues

This study was conducted according to the guidelines of the Declaration of Helsinki and all procedures involving human participants were approved by the Medical Ethics Review Board of Kanazawa University, Kanazawa, Japan (approval number 2018–129 (053)). The Ethics Review Board has waived the need for written informed consent and adopted that in case an individual returns the questionnaire, it is considered as consent. All data were obtained from the Hakui city government under an agreement paper related to the Academic Research and Privacy Protection. All data were completely anonymized before access and analysis. Consent for use of the dataset was granted by the municipal government of Hakui city after a formal application, along with an explicit pledge to protect the confidentiality of the data supplied.

### Self-rated health-related survey items

We measured self-rated health using the question, “How do you feel about your current health status: excellent, good, poor, or very poor?” The responses were classified as “Good self-rated health” for excellent and good, and “Poor self-rated health” for poor and very poor. Additionally, the self-rated health score (range, 1–4) was obtained based on the response as follows: one point for excellent, two points for good, three points for poor, and four points for very poor.

### Independent variables

At the individual level, we used age, gender, education, frequency of work, and family structure as independent variables. Age was coded into 20-year intervals and the education was grouped into four categories according to the duration (less than 10 years, 10–12 years, 13–15 years, and over 16 years). The frequency of work was grouped into three categories: working every day, working more than a few times a year, and not working. The family structure was grouped into four categories: families with two or more households, only a couple, living alone, and others.

Community social capital was assessed by cognitive and structural social capital. General trust and norm were adopted as independent variables for cognitive social capital. General trust was assessed using the question, “Generally speaking, do you think that most people can be trusted?” The general trust score (range, 0–2) was obtained as follows: two points for “strongly agree,” “mostly agree,” and “almost agree,” one point for “fair,” and zero points for “almost disagree,” “mostly disagree,” and “strongly disagree.” Regarding the norm, it was assessed with the questions [14], “Do you think everyone who lives in your neighborhood is a companion?” and “Do you want to be a useful person for your neighborhood?” The response options provided were as follows: “strongly agree,” “almost agree,” “almost disagree,” or “strongly disagree.” The responses were scored as follows: one point for “strongly agree” or “almost agree” and zero points for “almost disagree” or “strongly disagree.” The norm score (range, 0–2) was obtained by the sum of the items.

Structural social capital was measured using a question addressing each individual’s participatory level in the following three kinds of social events: local festivals, cleaning of local meeting houses and roads, and neighborhood association meetings. The response for each item was scored as follows: one point for “always participate” or “participate as much as possible” and zero points for “not much” or “never participated.” The civic participation score (range, 0–3) was obtained from the sum of the items.

Hakui city has 11 school districts. We considered school districts as a community unit because these were formerly villages that existed before a municipality merger took place in the 1950s. Social activities are often conducted within each school district, and people can easily walk or cycle within the school district where they live. Table 1 shows the characteristics of the residents in the 11 school districts. The lowest participation rate was 35.1% (school district 6) and the highest was 46.0% (school district 5).

**Table 1:** Characteristics of residents according to the 11 school districts.

School districts	Age (years)			Gender: Women N (%)	Population aged ≥65 years, %	Participants N (%)
	40–59 N (%)	60–79 N (%)	≧80 N (%)			
#1	1560 (36.5)	1963 (45.9)	753 (17.6)	2344 (54.8)	41.1	1873 (43.8)
#2	643 (38.3)	733 (43.7)	303 (18.0)	912 (54.3)	39.4	743 (44.2)
#3	576 (42.0)	589 (42.9)	207 (15.1)	741 (54.0)	35.4	572 (41.6)
#4	516 (41.2)	541 (43.2)	196 (15.6)	657 (52.4)	32.9	495 (39.5)
#5	675 (32.4)	989 (47.4)	421 (20.2)	1105 (52.9)	45.1	961 (46.0)
#6	86 (26.7)	157 (48.8)	79 (24.5)	178 (55.2)	53.6	113 (35.1)
#7	453 (32.6)	688 (49.6)	247 (17.8)	751 (54.1)	44.5	593 (42.7)
#8	112 (27.3)	208 (50.7)	90 (22.0)	221 (53.9)	49.0	185 (45.1)
#9	232 (28.2)	395 (48.1)	195 (23.7)	464 (56.4)	48.6	363 (44.1)
#10	329 (33.9)	462 (47.6)	180 (18.5)	524 (53.9)	43.8	446 (45.9)
#11	195 (29.4)	320 (48.2)	149 (22.4)	337 (50.7)	48.5	234 (35.2)

We aggregated individual responses into school districts to assess community-level social capital. Community-level social capitals (general trust, norm, and civic participation) were calculated from the mean of the social capital score of each community. The median household income of each community was obtained from Hakui city and adopted as a community variable.

### Statistical analyses

The age and gender of the respondents included in the final analysis were compared with those of the nonrespondents using the Chi-squared test. Participant individual characteristics were summarized for the prevalence of poor self-rated health. The demographic data and individual social capitals were compared across poor self-rated health using the Chi-squared test.

A multilevel analysis using a random intercept model was performed with data from 6578 individuals nested within the 11 communities in Hakui city. A multilevel linear regression model with maximum likelihood was used to estimate the relationship among self-rated health, community-level social capital, and other variables. First, we developed a null model, which was an empty model with no independent variables. The intra-class correlation coefficient was calculated to show the proportion of total variance that resides between communities at level 2. Second, we investigated the effects of individual social capital on self-rated health while controlling for demographic variables (Model 1). Third, we assessed the effects of community-level social capital on self-rated health while controlling for demographic variables (Model 2). Fourth, we assessed the association between community-level social capital and self-rated health after controlling for demographic and individual social capital (Model 3). Finally, Model 4 added the community-level socioeconomic variables to Model 3.

A multiple imputation analysis was applied to address the missing data. We used 10 chained equation analyses. For sensitivity analysis, we applied complete case analysis (n = 6279) after excluded participants with any missing value on the self-rated health and age.

A *P* < 0.05 was considered statistically significant. Furthermore, all statistical analyses were performed using the SPSS software (version 26; SPSS Inc., Chicago, IL, USA) and Amos.

## Results

The demographic characteristics of the respondents analyzed in the final analysis (n = 6578) included a higher proportion of subjects in their 60s and 70s (*P* < 0.001) and a higher proportion of women (*P* = 0.004) compared those of the nonrespondents (n = 8664). Low response rates were observed in the school districts with a high percentage of elderly (population aged ≥65 years). Of the 6578 participants, 1919 (29.1%) rated their health status as poor. Overall, the responses of the self-rated health were 14.4% (n = 950) for excellent (1 point), 56.4% (n = 3709) for good (2 points), 21.8% (n = 1431) for poor (3 points), and 7.4% (n = 488) for very poor (4 points). A higher proportion of self-rated poor health status was observed for older participants: 22.1%, 26.9%, and 46.6% for individuals in their 40s and 50s, 60s and 70s, and 80s and above, respectively (*P* < 0.001) (Table 2). A higher proportion of participants with a shorter education period self-rated their health status as poor (*P* < 0.001) (Table 2). Additionally, a lower level of individual social capitals (general trust, norm, and civic participation) indicated a higher level of self-rated poor health (*P* < 0.001, *P* < 0.001, and *P* < 0.001, respectively) (Table 2).

**Table 2:** Individual characteristics and prevalence of self-rated poor health.

Characteristics	N (%)	Self-rated poor health (%)
Age (years)		
40–59	1764 (26.8)	22.1
60–79	3649 (55.5)	26.9
≧80	1165 (17.7)	46.6
Gender		
Men	2929 (44.6)	30.2
Women	3649 (55.4)	28.3
Education (years)		
≤9	1468 (22.3)	40.8
10–12	2723 (41.4)	27.9
13–15	1311 (19.9)	24.0
≥16	1076 (16.4)	22.8
Frequency of work		
Every day	3139 (47.7)	21.1
More than a few times a year	1036 (15.7)	26.9
None	2403 (36.5)	40.6
Family structure		
Two or more households	1705 (25.9)	27.8
Only a couple	2168 (33.0)	28.2
Living alone	812 (12.3)	36.4
Others	1893 (28.8)	28.1
General trust score		
2 (highest trust)	2112 (32.1)	23.6
1 (middle trust)	3635 (55.3)	29.7
0 (lowest trust)	831 (12.6)	40.7
Norm score		
2 (highest norm)	3554 (54.0)	24.3
1 (middle norm)	1694 (25.8)	31.8
0 (lowest norm)	1330 (20.2)	39.4
Civic participation score		
3 (highest participation)	2523 (38.3)	20.5
2 (higher middle participation)	1232 (18.7)	26.9
1 (lower middle participation)	1056 (16.1)	29.9
0 (lowest participation)	1767 (26.9)	42.5

The intra-class correlation coefficient in the null model (n = 6578) for self-rated health was 2.420e^−3^. In Model 1 (n = 6578), after adjusting for demographic factors, the individual social capitals (general trust, norm, and civic participation) were significantly associated with self-rated health (*P* < 0.001, *P* < 0.001, and *P* < 0.001, respectively) (Table 3). Model 2 (n = 6578) demonstrated a significant association between community-level civic participation and self-rated health after adjusting for demographic factors (*P* = 0.043) (Table 3). This association remained unchanged even after adjustment for the individual social capitals (Model 3, n = 6578, *P* = 0.042). Model 4 (n = 6578) indicated that the community-level civic participation remained significant after adjusting for community-level household income in addition to Model 3 (*P* = 0.036) (Table 3). In Model 4, the community-level household income was also significantly associated with self-rated health (*P* = 0.007) (Table 3). No significant associations between community-level general trust, norm, and self-rated health were observed in the multilevel analysis. The sensitivity analysis performed using complete case analysis, after excluding participants with the missing values, revealed similar results (S1 Table). The variability between and within school districts were 1.188 (P = 0.237) and 2.867e^-3^ for general trust, 1.087 (P = 0.283), 3.600e^-3^ for norm, and 1.181 (P = 0.238), 1.500e^-3^ for civic participation.

**Table 3:** Multilevel logit estimates for reporting self-rated poor health with multiple imputation.

	Model 1 (n = 6578)		Model 2 (n = 6578)		Model 3 (n = 6578)		Model 4 (n = 6578)	
	Estimates (SE)	*P*	Estimates (SE)	*P*	Estimates (SE)	*P*	Estimates (SE)	*P*
Level 1 (Individual)								
Age (years) (ref. ≧80 years)								
40–59	−0.258 (0.033)	<0.001	−0.230 (0.033)	<0.001	−0.258 (0.033)	<0.001	−0.257 (0.033)	<0.001
60–79	−0.189 (0.026)	<0.001	−0.235 (0.026)	<0.001	−0.190 (0.026)	<0.001	−0.190 (0.026)	<0.001
Education (years) (ref. ≥ 16 years)								
≤9	0.145 (0.031)	0.001	0.191 (0.032)	0.001	0.148 (0.031)	0.001	0.143 (0.031)	<0.001
10–12	0.079 (0.026)	0.003	0.097 (0.027)	0.001	0.080 (0.026)	0.003	0.079 (0.026)	0.003
13–15	0.058 (0.030)	0.057	0.073 (0.031)	0.194	0.058 (0.030)	0.058	0.057 (0.030)	0.062
Men (ref. Women)	0.138 (0.019)	<0.001	0.079 (0.019)	<0.001	0.134 (0.019)	< 0.001	0.134 (0.019)	<0.001
Frequency of work (ref. None)								
More than a few times a year	−0.199 (0.027)	<0.001	−0.232 (0.028)	<0.001	−0.196 (0.027)	<0.001	−0.199 (0.027)	<0.001
Every day	−0.289 (0.022)	<0.001	−0.321 (0.023)	<0.001	−0.289 (0.022)	<0.001	−0.290 (0.022)	<0.001
Family structure (ref. Living alone)								
Only a couple	−0.067 (0.030)	0.034	−0.074 (0.031)	0.024	−0.065 (0.030)	0.039	−0.065 (0.030)	0.039
Two or more household	−0.027 (0.031)	0.389	−0.049 (0.090)	0.138	−0.024 (0.031)	0.434	−0.052 (0.031)	0.556
Others	−0.019 (0.031)	0.552	−0.017 (0.032)	0.572	−0.018 (0.031)	0.575	−0.052 (0.031)	0.556
General trust	−0.055 (0.009)	<0.001			−0.085 (0.014)	<0.001	−0.085 (0.014)	<0.001
Norm	−0.056 (0.014)	<0.001			−0.064 (0.012)	<0.001	−0.064 (0.012)	<0.001
Civic participation	−0.095 (0.009)	<0.001			−0.110 (0.008)	<0.001	−0.110 (0.008)	<0.001
Level 2 (Community)								
Average household income							−0.024 (0.008)	0.007
General trust			−0.111 (0.341)	0.679	−0.084 (0.338)	0.693	−0.262 (0.300)	0.434
Norm			−0.080 (0.291)	0.754	−0.111 (0.288)	0.706	0.098 (0.265)	0.586
Civic participation			−0.201 (0.084)	0.043	−0.202 (0.084)	0.042	−0.163 (0.072)	0.036

## Discussion

We demonstrated that community-level civic participation was negatively associated with self-rated poor health in a rural town where the population was declining and aging. In contrast, community-level general trust and norm were not significantly associated with self-rated health. These findings highlight the importance of strategies to improve the health of adults and older individuals through fostering civic participation in communities.

Community-level civic participation estimate was not substantially altered by the inclusion of the individual level social capital measures. This implied that the civic participation measures on the two levels did not correlate. Our results are consistent with the contextual effect of civic participation on self-rated health independent of individual-level civic participation. Living in high civic participation communities may result in a decreased risk for poor self-rated health independent of individual-level civic participation. In the present study, we surveyed the residents’ level of participation in large-scale events, such as local festivals, cleaning of local meeting houses and roads, and neighborhood association meetings. In contrast to our results, it has been reported that self-rated health is associated with community-level cognitive social capital, such as trust and reciprocity, rather than with community-level structural social capital, such as civic participation [3, 4] and daily interaction with others [4]. One possible reason for the discrepancy between our results and those of the previous study could be attributed to the difference in participation types. Hibino et al. analyzed civic participation using a questionnaire on individual participation in eight kinds of social groups, such as political associations, trade associations, and so on [3]; Inaba et al. analyzed it using a questionnaire on individual participation in group activities, such as community, volunteer, and sports [4]. In their studies, the residents’ participation level was analyzed based on their participation in small- or medium-scale events as the number of people participating was smaller than our study wherein the number of participants was large, resulting into a large scale event. Some types of participation may propagate detrimental health behaviors and contribute to chronic stress from increased costs of participants [5]. If different forms of civic participation were used in the survey, the association between the community-level civic participation and the individual level of self-rated health might differ. In addition, no association between civic participation and health has been reported in several longitudinal cohort studies in urban areas [5, 6]. Future longitudinal studies comparing rural and urban areas are needed to explore the relationship between community-level civic participation and self-rated health in rural areas.

Regarding the association between community-level civic participation and health other than self-rated health, longitudinal cohort studies have demonstrated that baseline civic participation is associated with better health outcomes, such as a reduced risk of mortality [7, 8], reduced risk of cognitive impairment [8], and prevention of functional disability [9].

However, with regard to the community-level general trust and norm, our results did not support previous findings, unexpectedly. Individual general trust and norm were significantly associated with self-rated health, whereas at the community level, no significant associations were observed among general trust, norm, and self-rated health. Several nationwide studies conducted in Japan or the United Kingdom have found that community-level cognitive components, such as general trust and norm, are associated with better self-rated health [3–5]. However, in the studies conducted in South Africa, Chola et al. reported that no association was found between individual general trust and self-rated health [15], and Lau et al. reported that higher levels of contextual general trust were associated with poor self-rated health [16]. It is difficult to compare this association, with regard to social capital, with other studies because social capital has been conceptualized differently. The conflicting results regarding the association between general trust and health may be due to the differences in the way this variable has been constructed between studies. Our study was conducted in a town where population decline and aging had progressed. Because of depopulation and aging, many residents are unhealthy, resulting in decline in the cognitive social capital with no positive effects.

We showed that community household income level was significantly associated with self-rated health. Similar to our results, it was reported that the individual level of household income can impact health such as self-rated health [3], psychological distress [17], and functional disability [18]. Additionally, it was reported that an increased Gini coefficient was negatively associated with community-level social capital [19] and a decreased Gini coefficient was associated with good self-rated health [19]. This study may provide additional evidence to support a pathway between community-level income inequality and health.

The present study had several limitations. First, this study was cross-sectional design in nature. Therefore, it is not possible to make causal attributions or discern the direction of the relationship between social capital and self-rated health. Future longitudinal studies are needed to explore the direction of the relationship between social capital and self-rated health. Second, because the measurement was based on a self-administered questionnaire, the results are subject to response biases. Third, as the response rate of the survey was low (43.2%), individuals excluded from the analyses were more likely to be men and aged 40–59 or ≥80 years. Additionally, low response rates were observed in the school districts with a high percentage of elderly.

This study has some limitations. Therefore, we should be cautious in drawing any specific conclusions. However, our findings might demonstrate the importance of fostering communities with high civic participation to reduce the poor health status of residents.

## Supporting information

S1 Table Results of the sensitivity analysis (complete case analysis). Multilevel logit estimates for reporting self-rated poor health.
(DOCX)
