# Supplementary material for: Residents living in communities with higher civic participation report higher self-rated health
Source: PLoS One. 2020 Oct 23;15(10):e0241221. doi: 10.1371/journal.pone.0241221 (PMC7584176; doi:10.1371/journal.pone.0241221)
Supplement: S1 Table — Multilevel logit estimates for reporting self-rated poor health. (DOCX) [file pone.0241221.s001.docx]

**S1 Table. Results of the sensitivity analysis (complete case analysis). Multilevel logit estimates for reporting self-rated poor health**

|  | Model 1 (n = 4642) | | Model 2 (n = 4930) | | Model 3 (n = 4642) | | Model 4 (n = 4642) | |
| --- | --- | --- | --- | --- | --- | --- | --- | --- |
|  | Estimates (SE) | *P* | Estimates (SE) | *P* | Estimates (SE) | *P* | Estimates (SE) | *P* |
| Level 1 (Individual)  Age (years) (ref. ≧80 years)  40–59  60–79  Education (years) (ref. ≥ 16 years)  ≤9  10–12  13–15  Men (ref. Women)  Frequency of work (ref. None)  More than a few times a year  Every day  Family structure (ref. Living alone)  Only a couple  Two or more household  Others  General trust  Norm  Civic participation  Level 2 (Community)  Average household income  General trust  Norm  Civic participation | −0.245 (0.045)  −0.221 (0.038)  0.131 (0.038)  0.065 (0.029)  0.061 (0.033)  0.152 (0.022)  −0.217 (0.032)  −0.296 (0.027)  −0.094 (0.039)  −0.034 (0.038)  −0.012 (0.059)  −0.055 (0.009)  −0.056 (0.014)  −0.095 (0.009) | <0.001  <0.001  0.001  0.024  0.064  <0.001  <0.001  <0.001  0.016  0.370  0.843  <0.001  <0.001  <0.001 | −0.219 (0.043)  −0.256 (0.036)  0.193 (0.037)  0.088 (0.029)  0.069 (0.033)  0.093 (0.021)  −0.257 (0.032)  −0.340 (0.027)  −0.098 (0.038)  −0.036 (0.037)  0.020 (0.058)  −0.246 (0.166)  0.274 (0.292)  −0.187 (0.070) | <0.001  <0.001  0.001  0.024  0.064  <0.001  <0.001  <0.001  0.010  0.327  0.722  0.139  0.346  0.008 | −0.247 (0.045)  −0.221 (0.038)  0.133 (0.038)  0.065 (0.029)  0.060 (0.033)  0.152 (0.022)  −0.214 (0.032)  −0.294 (0.027)  −0.092 (0.039)  −0.031 (0.037)  −0.007 (0.059)  −0.055 (0.009)  −0.056 (0.015)  −0.095 (0.009)  −0.227 (0.184)  0.327 (0.318)  −0.218 (0.078) | <0.001  <0.001  0.001  0.025  0.067  < 0.001  <0.001  <0.001  0.019  0.396  0.905  <0.001  <0.001  <0.001  0.263  0.333  0.030 | −0.244 (0.045)  −0.221 (0.038)  0.126 (0.038)  0.064 (0.029)  0.059 (0.032)  0.152 (0.022)  −0.218 (0.032)  −0.296 (0.027)  −0.093 (0.039)  −0.036 (0.037)  −0.013 (0.059)  −0.055 (0.097)  −0.056 (0.014)  −0.096 (0.009)  −0.028 (0.009)  −0.247 (0.166)  0.464 (0.296)  −0.194 (0.071) | <0.001  <0.001  <0.001  0.003  0.102  <0.001  <0.001  <0.001  0.017  0.343  0.819  <0.001  <0.001  <0.001  0.003  0.137  0.118  0.006 |
